# Reported Agonistic Behaviours in Domestic Horses Cluster According to Context

**DOI:** 10.3390/ani14040629

**Published:** 2024-02-16

**Authors:** Kate Fenner, Bethany Jessica Wilson, Colette Ermers, Paul Damien McGreevy

**Affiliations:** 1 School of Agriculture and Food Science, The University of Queensland, Gatton, QLD 4343, Australia; kate.fenner@uq.edu.au; 2 School of Life and Environmental Science, The University of Sydney, Sydney, NSW 2006, Australia; 3 School of Environment and Rural Science, University of New England, Armidale, NSW 2351, Australia; colette.ermers@gmail.com; 4 Sydney School of Veterinary Science, The University of Sydney, Sydney, NSW 2006, Australia

**Keywords:** aggression, defence, distance-increasing behaviour, resource-guarding

## Abstract

**Simple Summary:**

Horses respond to threats by moving away or, if that is not an option, by behaving in ways that deter the threat or move it away. These responses that help to reduce the threat are regularly labelled aggression, but this overlooks the motivation that underpins the responses which are often simply forms of defence. A catch-all term that avoids the need to interpret the motivation for defence and aggression responses is agonistic behaviour. In domestic horses, all such responses are important because they can be dangerous for both horses and personnel; they can prompt humans to defend themselves and can compromise the relationships that horses have with their carers. This study used an online questionnaire to ask owners about the management, training, and behaviour of the horses in their care. Although this questionnaire included data on horses that had not yet been trained to be ridden or had retired, this article focuses on the results of 2743 ridden horses. It reports a scale from warning signs—threatening to bite, pinning ears, tail swishing, threatening to kick or strike—to the most serious actions that include actual biting, kicking, or striking. The analysis revealed that agonistic behaviour is associated with certain management or training activities and that horses that show one form of behaviour are likely to show others in the same context. This means that some horses are particularly motivated to show these responses by certain triggers that arise in such contexts. The clusters with common characteristics were those observed in the context of: accelerating when ridden; saddling; familiar management activities; proximity to unfamiliar horses and other species. Taken together, these results highlight the prospect that the motivation to show these responses differs with context. This finding challenges the simplistic view that the problems with equine aggression and defence lie with the horses themselves rather than with historic or current management practices.

**Abstract:**

Agonistic behaviours are often directed at other animals for self-defence or to increase distance from valued resources, such as food. Examples include aggression and counter-predator behaviours. Contemporary diets may boost the value of food as a resource and create unanticipated associations with the humans who deliver it. At the same time the domestic horse is asked to carry the weight of riders and perform manoeuvres that, ethologically, are out-of-context and may be associated with instances of pain, confusion, or fear. Agonistic responses can endanger personnel and conspecifics. They are traditionally grouped along with so-called vices as being undesirable and worthy of punishment; a response that can often make horses more dangerous. The current study used data from the validated online Equine Behavioural and Research Questionnaire (E-BARQ) to explore the agonistic behaviours (as reported by the owners) of 2734 horses. With a focus on ridden horses, the behaviours of interest in the current study ranged from biting and bite threats and kicking and kick threats to tail swishing as an accompaniment to signs of escalating irritation when horses are approached, prepared for ridden work, ridden, and hosed down (e.g., after work). Analysis of the responses according to the context in which they arise included a dendrographic analysis that identified five clusters of agonistic behaviours among certain groups of horses and a principal component analysis that revealed six components, strongly related to the five clusters. Taken together, these results highlight the prospect that the motivation to show these responses differs with context. The clusters with common characteristics were those observed in the context of: locomotion under saddle; saddling; reactions in a familiar environment, inter-specific threats, and intra-specific threats. These findings highlight the potential roles of fear and pain in such unwelcome responses and challenge the simplistic view that the problems lie with the nature of the horses themselves rather than historic or current management practices. Improved understanding of agonistic responses in horses will reduce the inclination of owners to label horses that show such context-specific responses as being generally aggressive.

## 1. Introduction

Agonistic behaviour is any social interaction that involves fighting, placation, and conciliation [1]. Being either offensive or defensive, it has broader meaning than simple aggression because it includes threats, displays, displacement, retreats, and deference. In horses, agonistic behaviours can include avoidance, aggression, and counter-predator behaviours. The current study focuses on undesirable behaviours directed at others who are either familiar or unfamiliar. These others include horses, humans, and other species. In its broadest sense, the term avoids inferring that the motivation for such responses is either aggression or defence.

Agonistic behaviours are of importance to horse owners for at least three reasons: they are dangerous to personnel, can compromise the horse–human bond, and can affect the market value of horses. These outcomes can have profound implications for horse welfare because undesirable behaviours are often misinterpreted as lack of so-called respect and can initiate punitive actions by personnel [2]. Unfortunately, as with other animals and humans, punishment (intended to delete undesirable behaviours) in horses is rarely well timed or salient enough to be effective in reducing the motivation behind the behaviour. As a result, there is a risk of the persistent unwelcome behaviour prompting some humans to escalate the force of the aversive stimuli they apply. In some instances, it is possible that these consequences may lead to violence that can increase the arousal of the horse, send them into a negative affective state, and make them more fearful of humans. This, in turn, makes them more dangerous to handle and further compromises the human–horse bond [3].

From an equine welfare perspective, the most important cause of agonistic responses is pain, which is often undiagnosed [4]. Pain associated with being handled and ridden (potentially with flawed techniques) is thought to prompt some horses to be aggressive and motivate them to increase the distance between themselves and humans [5]. For example, most horses severely affected by vertebral problems (33/43 horses in a sample of riding horses) were prone to react aggressively towards humans, which was not the case for comparable unaffected or slightly affected horses [6]. Signs of irritation when ridden and handled also often reflect pain that is heightened when bearing the weight of a rider. Also, the common use of pressure-based cues in equitation can signal horses to undertake athletic manoeuvres that, because of pain, some would prefer to avoid. These responses can be learned because they may dislodge riders and, thus, become negatively reinforced by the cessation of pressure [7].

When food is the resource being guarded by agonistic behaviour, it makes sense to consider those attributes of that food that make it valuable to horses and therefore worth defending. Acquisition of a highly valued resource can explain why horses learn to be aggressive around food so the learned aspect of food-related aggression merits consideration. It would seem logical that diets that are high in starch, such as prepackaged concentrated feeds, may be high-risk for learned aggression because they are made to be palatable and are expected to be of high hedonic value to the horse and, therefore, worth the risk of an unwelcome outcome (such as triggering a defence response from protagonists) [8]. The same principle holds for all foods that are provided as discrete meals, in that limited access may heighten the perceived value of the food.

Horses may associate certain parts of their environment (such as feeding stations and mangers) with high-value food and deter horses and even personnel from approaching them [9]. Again, this can be misinterpreted by personnel as random aggression and can attract punishment, which morphs the motivation to aggress into self-defence and generally escalates the efforts of both members of the dyad. Other notable triggers for agonistic responses may reflect a motivation to keep unfamiliar conspecifics at a distance and to protect offspring.

It is important to recognise that various triggers may arise in combination. For example, horses on concentrated diets tend to be those in work and, given that different equestrian disciplines have different energy demands, there is likely to be interaction between discipline and diet. Also, it is logical that pastured-based diets are associated with less confinement than concentrate-based diets typical of stabled horses. Given that multiple variables exist in horses’ lives at the same time, we decided to use a survey of owners to unpack the various contexts in which agonistic responses arise. The current study explores relationships between feeding practices, the disciplines horses are being trained for, and undesirable behaviours as reported by owners. The behaviours of interest ranged from biting and bite threats and kicking and kick threats to tail swishing as an accompaniment to signs of escalating irritation when horses were approached, prepared for ridden work, ridden, and hosed down (e.g., after work). The study used owners’ reports through a validated behavioural questionnaire to explore how the management of horses varies across disciplines and how the reported agonistic behaviours cluster together.

## 2. Materials and Methods

### 2.1. Survey

The project was approved by the University of Sydney Human Research Ethics Committee (approval number: 2020/326). The Equine Behavior Assessment and Research Questionnaire (E-BARQ), designed to draw objective data, is a not-for-profit, validated, equine behavioural questionnaire created on the Qualtrics platform [Qualtrics]. The questionnaire consists of 97 matrix-style questions, which include 42 demographic items about the horse owner and handler. The questionnaire then divides into 268 items for the ridden or driven horse and 218 items for the non-ridden horse [10] (see Supplementary File S1). E-BARQ is an ongoing project and, for the purposes of the current study, was distributed to horse owners, riders, and trainers through social media platforms such as Facebook and Instagram. It was distributed via social media, equestrian sport organisations’ email contacts, and the email lists of *Horses and People Magazine* (https://horsesandpeople.com.au/; accessed on 1 September 2023), *Equitation Science International* (https://www.esi-education.com/; accessed on 1 September 2023), and *Kandoo Equine* (https://www.kandooequine.com/; accessed on 1 September 2023).

Survey respondents were 76.96% female and 5.16% male (with 17.89% choosing not to answer the question, indicating they would prefer not to say or indicating they were neither male nor female). The age distributions of the respondents were 3.95% <18 years; 19.82% 18–24 years; 14.84% 25–34 years; 12.61% 35–44 years; 14.63% 45–54 years; 12.83% 55–64 years old; 3.99% 65–74 years; 0.22% >75 years old; and 17.11% unknown.

E-BARQ is a longitudinal study and horse owners are invited to update their responses every six months. For the purposes of this study, where one horse has been reported on sequentially over time (10% of respondents), only the first report was included in the current analysis. E-BARQ specifies that it welcomes responses from respondents who are familiar with the focal horse’s behaviour, with the following statement: “You have been invited to participate in this study because you are a horse owner and familiar with your own horse’s behaviour”.

### 2.2. Analysis

Statistical analysis was undertaken using R version 4.4.2 [11]. Owner reports of agonistic behaviours were examined in both the unridden (22 items) and ridden (29 items) horses in the E-BARQ responses. Items included behaviours exhibited when the horse was corrected, approached, saddled, handled, in the company of familiar and unfamiliar other animals and humans, and signalled to move. Respondents scored responses in each of the 29 circumstances on an ordinal scale from 0 (no signs) to 4 (serious signs, e.g., bite, kick, strike). The underlying structure of the agonistic behaviour data was explored by a hierarchical cluster analysis based on Gower’s general dissimilarity coefficient, due to the ordinal nature of the data, and a principal component analysis with varimax rotation. Calculation of the dissimilarity matrix was undertaken using the cluster package of R [12]. and a dendrogram was assembled by agglomerative hierarchical clustering using the Ward’s minimum variance method, implementing Ward’s clustering criterion. A principal component analysis (PCA) of the scores was undertaken to further explore the underlying structure of the aggression data and thus identify its rotated components (RC) using the psych package [13]. The number of components to be retained was determined by considering parallel analysis, the scree plot, and the Kaisar rule. The retained components were rotated using the orthogonal ‘varimax’ rotation.

E-BARQ notes that: “Some horses display defensive or aggressive behaviour in certain situations. Typical signs would include threatening to bite, pinning ears, tail swishing, threatening to kick or strike. The most serious signs would include actual biting, kicking or striking.” Respondents were then directed as follows: “Check a box on the 5-point scale below to indicate your horse’s recent tendency (using the previous 6 months as a guide) to show these behaviours.”

The 29 circumstances in which E-BARQ respondents can report agonistic behaviours by their (focal) horses were when their horses were: *verbally corrected by owner on ground*; *verbally corrected when ridden/driven*; *given leg or whip cues when ridden/driven*; *approached by owner in paddock*; *approached by owner when tied*; *approached by owner in stall*; *approached by stranger in paddock*; *approached by stranger in stall*; *approached by stranger in paddock*; *approached by owner when eating*; *approached by owner carrying feed*; *[having their] saddle placed on back*; *[having their] girth done up*; *hosed down*; *approached by familiar dog*; *approached by familiar animal*; *approached by unfamiliar dog*; *approached by unfamiliar animal*; *led toward unfamiliar horse*; *approached by unfamiliar horse*; *ridden/driven toward unfamiliar horse*; *led beside unfamiliar horse*; *ridden/driven beside unfamiliar horse*; *ridden/driven in a group*; *ridden/driven in arena with other horses*; *lunged in a round pen*; *signalled forward under saddle*; *signalled to canter under saddle*; and *signalled to increase speed under saddle*. If none of these applied, the respondent selected either “Never” or “Not applicable”.

## 3. Results

A total of 5721 records were supplied for 3391 horses (2734 ridden and 657 unridden). There were 1942 geldings (57.3%), 1275 mares (37.6%), 67 stallions (2.0%), 62 fillies (1.8%), 16 colts (0.7%), 6 rigs (0.2%), and 23 horses for which the question was not answered (0.7%). In summary, the types of equids were crossbreds (*n* = 1060), purebred draft breeds (*n* = 27), purebred light breeds (*n* = 1428), purebred pony breeds (*n* = 105), and unspecified purebreds (*n* = 114).

The respondents were from Africa (*n* = 25), Asia (16), Europe (567), North America (681), Oceania (968), South America (11), and of unstated region (466).

The results of the analyses are reported in terms of the intensity of signs of aggression and the underlying structure of the aggression data.

### 3.1. Intensity of Signs of Aggression

**Figure 1:**
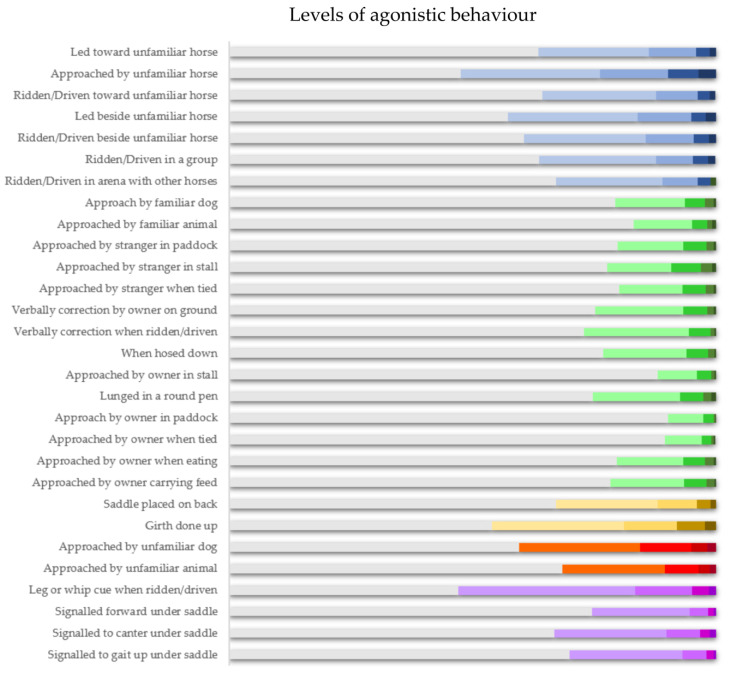
The distribution of unwelcome responses among the ridden horses (*n* = 2734) reported by owners in the current study. The rows show the proportion of horses reported to show responses of various intensity (as reported from no signs, with no colour, to serious signs, with darkening colours). The rows are shaded to indicate the gravity of the signs and are highlighted with colours that reflect the emergent dendrographic clusters (see below) as follows: intra-specific threats (blue); inter-specific threats (red); extraneous disturbances in a familiar environment (green); saddling-up (yellow); and locomotory effort (purple).

Among the 2734 ridden horses reported on by owners in the current study, the intensity of agonistic responses was reported from 0 to 4. The distribution of the reported intensity of these responses appears in Figure 1.

The context in which agonistic responses were most frequently not observed (Intensity 0) was when the focal horse was approached by the owner in the paddock (90.2% of the sample). Conversely, the circumstance that most was frequently associated with the most severe signs (Intensity 4) was when the focal horse was approached by an unfamiliar horse (3.6% of the sample) and when the girth was fastened (2.3%).

Of the horses that showed any signs of agonistic behaviour (Intensity 1–4), the girth being fastened was again commonly reported, with 46% of all horses showing some signs. However, the most common context was when they received a leg or whip cue when ridden/driven (53% of the sample). When this was revisited with related questions, owners reported signs in 33.2% of horses when signalled to canter under saddle, 30% of horses when signalled to gait up under saddle, and 25.4% when signalled forward under saddle. Meanwhile, 25.3% did so when lunged.

### 3.2. Underlying Structure of the Aggression Data

The underlying structure of the aggression data of the ridden horses (as distinct from those that were yet to be ridden) was explored using two methods: cluster analysis, which groups observations according to their dissimilarity, and (varimax rotated) principal component analysis, which finds a smaller set of artificial variables (components) and rotates them to improve interpretability.

#### 3.2.1. Clusters of Agonistic Behaviour According to Context

**Figure 2:**
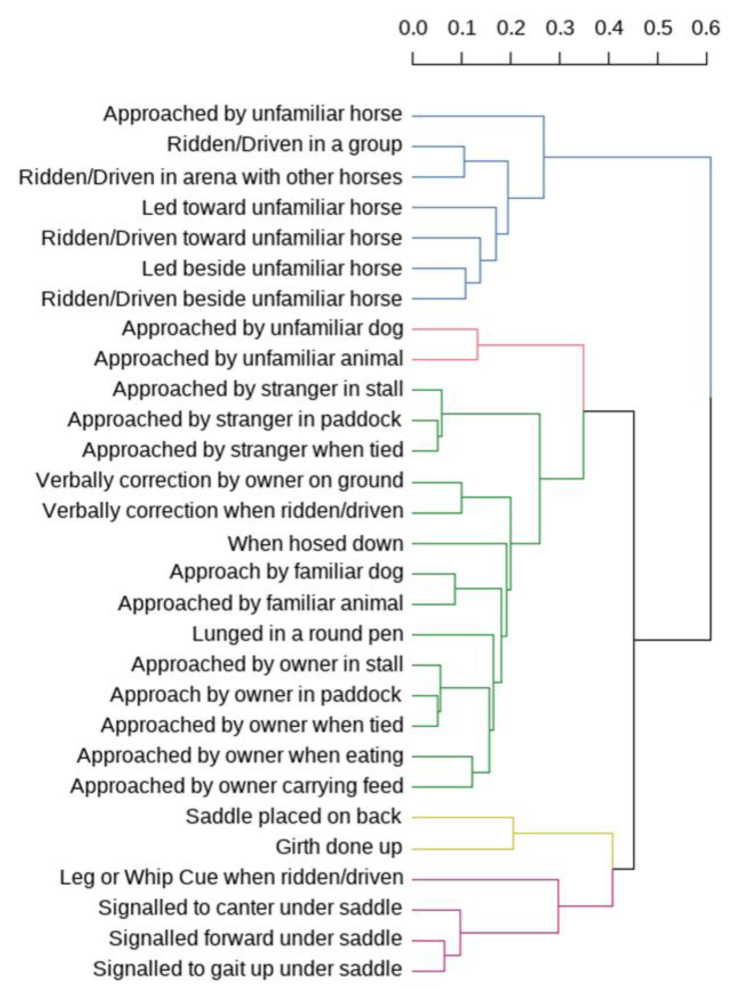
Hierarchical cluster dendrogram for the reported aggression scores of the ridden horses showing five clusters (coloured blue, red, green, yellow, and purple) within the structure of the dissimilarity matrix for the 29 potential aggression triggers. The clusters reveal horses whose thresholds for agonistic behaviour vary with: intra-specific threats (blue); inter-specific threats (red); extraneous disturbances in a familiar environment (green); saddling-up (yellow); and locomotory effort (purple).

The hierarchy of dissimilarity across the contexts for the reported aggression scores of the ridden horses revealed five distinct clusters that we have labelled according to their common characteristics: locomotion under saddle; saddling; reactions in a familiar environment, inter-specific threats, and intra-specific threats. A dendrogram based on hierarchical clustering of the dissimilarity matrix of the 29 contexts that trigger the agonistic responses appears in Figure 2.

Considering the 1061 horses for which we have a complete set of aggression scores, 58.2% showed at least some agonistic responses to at least one of the seven intra-specific threat (blue cluster) situations; 37.4% showed at least some agonistic responses at least one of the two inter-specific threat (red cluster) situations; 60.7% showed at least some agonistic responses to at least one of the 13 extraneous disturbances in a familiar environment (green cluster) situations; 47.4% showed at least one of the two agonistic responses in the context of saddling (yellow cluster) situations; and 52.7% showed at least some of the four agonistic responses during locomotory effort (purple cluster) situations.

#### 3.2.2. Principal Component Analysis

**Figure 3:**
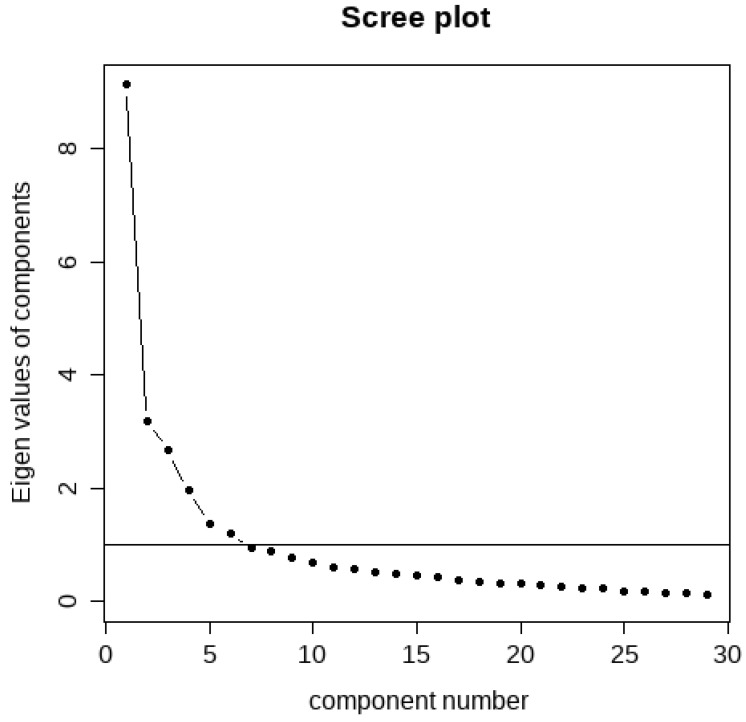
Scree plot of all of the components in the principal comments analysis and threshold for inclusion according to the eigenvalue of 1.

Parallel analysis suggested the extraction of six components, which was consistent with the scree plot (see Figure 3) and Kaiser rule.

The extracted principal components were subsequently rotated using a varimax rotation. These six components in Table 1 explain 0.67 of the variance.

Rotated component 1, accounting for 18% of the variance, corresponded to intra-specific threats (blue cluster) including the same seven items which formed part of this cluster. Rotated component 3, accounting for 13% of the variance, included all items from locomotory effort (purple cluster) but also included within this component were three items from the familiar environment cluster. Rotated clusters 4 and 5 displayed a similar pattern to rotated cluster 3, each containing the items of a cluster (the inter-specific threats and saddling up clusters, respectively) along with a smattering of familiar environment items. RC2 and RC6 included familiar environment items only. Therefore, while the PCA was supportive of the existence of four of the identified clusters, support for the fifth (familiar environment) cluster was more equivocal.

## 4. Discussion

The cluster analysis of dissimilarity revealed five distinct clusters with common characteristics: locomotion under saddle; saddling; reactions in a familiar environment, inter-specific threats, and intra-specific threats. While the PCA was supportive of the existence of four of the identified clusters, support for the fifth (familiar environment) cluster was more equivocal. Specifically, it broadly supported the dendographic organization, although some contextual triggers that clustered with extraneous disturbances in a familiar environment (green cluster) loaded onto other components and the remaining contexts loaded (or cross-loaded) onto two components (RC2 and RC6). We shall discuss the five clusters to explore possible underlying motivations, noting that motivating triggers often relate to fear and pain.

Of the current sample, 52.70% of the 1061 horses showed at least some agonistic responses during locomotory effort (purple cluster) situations. The four items in this cluster (*given leg or whip cues when ridden/driven*; *signalled to canter under saddle*; and *signalled to increase speed under saddle*, and *signalled forward under saddle*) are so closely linked in practice that any influences on the affective state of the horses are virtually indistinguishable. That said, the items have slight differences, but all reveal responses to the rider’s signalling for more muscular effort or speed. These responses may be associated with conflicting signals and frustration, but may also cause increased musculoskeletal pain as a product, for example, of landing with more force [14]. These results align with the findings of increased conflict behaviours and salivary cortisol concentrations in show-jumping horses with greater jumping faults and increased course difficulty [15]. These items were included in E-BARQ to ensure that cues, transition to canter, upwards transitions, and general forward locomotion could be scrutinized separately. Here they represent triggers for what many equestrian manuals refer to as resistance, and include conflict behaviours such as baulking, falling out of a gait, bucking, and rearing [9]. We acknowledge that these could be learned responses to inconsistent cues as part of flawed training but the need to eliminate pain before assuming the affected horses are simply being “lazy” or “stubborn” is critical.

Locomotion under saddle, the horse’s responses to the rider’s signals, and the absence of conflict behaviour arguably define the most important attributes of the ridden horse [16]. This cluster of items contribute to the horse’s perceived ‘rideability’ and, as such, often define its usefulness and thus dictate its welfare in its everyday life. Winther Christensen et al. [17] found that conflict behaviour levels were inversely related to ‘rideability’ scores and that riders impacted rein tension levels, horses’ heart rate, and salivary cortisol concentrations in dressage horses. A further rider effect was found in the tendency to display certain conflict behaviours, indicating rider-induced discomfort or confusion leading to associated welfare risks [17].

Aggressive riding behaviour has also been found to increase conflict behaviours with horses in the competitive sport of barrel racing [18]. Aggressive riding, which included both the use of the whip and the leg in an effort to increase horse speed, was not found to improve performance. However, horses that were whipped more were also more likely to react poorly to entering the arena and horses that were kicked more were found to be more likely to perform small rears, a behaviour that was also significantly associated with tail swishing [18].

Luke et al. [19] assessed horse behaviour and welfare using an online self-assessment survey. They found that horse welfare and ridden hyperactivity scores were significantly negatively related; meaning that, as welfare scores increased, the presence of hyperactive behaviours decreased. The same relationship was found between horse welfare scores and reported rider accidents and injuries, with horse behaviour being cited as the most commonly reported cause (75%) [19]. The relationship between rider safety and horse behaviour and welfare is becoming increasingly clear and E-BARQ, as a freely available behavioural monitoring tool, has the potential to improve rider safety and horse welfare across all riding disciplines.

Of the current sample of 1061 horses, 47.4% showed at least some agonistic responses in the context of saddling (yellow cluster). This means these two responses were seen in fewer horses than those in the other four clusters. Horses that predict pain when the saddle is placed on their back or the girth is tightened have historically been labelled cold-backed. The traditional approach to these cases has been to tighten the girth slowly and use pads (also known as numnahs) under the saddle to reduce the acute imposition of the gear [20]. Neither of these approaches does anything to address the causal pathology or saddle-fit deficit that is causing the pain.

Saddle fit has historically been assessed manually and visually, but this subjective procedure can lead to disagreement, even between qualified saddle fitters [21]. Dittmann et al. [22] investigated the prevalence of saddle-fit problems in Swiss riding horses and found only 10% of fitted saddles were free from problems, with pressures exceeding clinically relevant thresholds in 15% of horses; all despite most owners claiming their saddle was an ideal fit for their horse.

Dyson et al. [23] noted a relationship between equine behaviour when tacked-up and mounted and lameness. Specifically, tight tree points in the saddle and epaxial muscle pain were associated with higher behaviour scores during tacking up. This helps to explain the close dendrographic relationship between agonistic responses during saddling and those that cluster in response cues for locomotory effort when ridden (see above).

Of the current sample of 1061 horses, 60.7% showed at least some agonistic responses to one or more of the extraneous disturbances in a familiar environment (green cluster). These responses, largely to human disturbance in the paddock and stall or when tied-up, were seen in more horses than those in the other four clusters. However, this finding may simply reflect the cluster including more contexts than the others. Elements of E-BARQ that focus on how often horses respond with agonistic behaviours to grooming and clipping will be the focus of a separate report and may reveal influences on the reported ticklishness of horses. However, the current study shows that being hosed down (after work) and being lunged elicit agonistic responses but, because they did not cluster closely together, it seems likely that such responses have distinct motivations.

Hosing down may inadvertently impose a thermal challenge resulting in agonistic responses or it may simply reflect sensitivity to tactile stimuli or the presence of a handler. Interestingly, Hintze et al. [24] found qualitative observations revealed no difference between horses’ responses when being groomed or with the experimenter simply making the grooming movements without touching the horse. Their findings suggest that behaviours reported among the current findings are due to the tactile stimuli rather than the proximity of the handler. Lunging in a round pen, the other item in this cluster, may involve chasing or forcing the horse to perform beyond its fitness or pain threshold, resulting in these behaviours [2].

It is worth noting that agonistic responses to verbal correction clustered together. This merits further investigation to determine whether it is the correction or the possible accompanying use of whips or change in prosody that triggers the response [25]. Understanding the role of vocal characteristics as we engage with horses is a poorly studied area [26].

As these contexts for aggression did not cluster together, horses seem to distinguish between owners and unfamiliar people when responding to these signals in the contexts of paddocks, stalls, and when tied-up. That said, the severity of responses to either group of humans (see Figure 1) is comparable, so there is no evidence here that horses escalate responses to either group. Elements of E-BARQ that focus on how horses respond to veterinarians and farriers will be the focus of a separate report.

It is worth noting the close and distinct association between agonistic responses and the presence of food. (i.e., as observed when horses are approached by the owner when the human is carrying feed or the horse is eating). Having evolved to graze and browse on resources that are dispersed, horses show little food-related aggression in the free-ranging state [27]. In contrast, the content and presentation of rations to domestic horses are often characterised by discrete, focal meals, not the continuous trickle feeding they have evolved for. Delivering such focal meals has also been found to increase the horses’ motivation to search for food [28]. Different equestrian disciplines tend to be associated with particular management practices around feeding, housing, time spent at pasture, and exercise in equine company. The relationship between diet and behaviour may have profound implications for horse welfare and rider safety. For example, plasma serotonin concentrations were significantly higher in horses fed a high-fibre diet than in those fed a high-starch diet [29]. So, diet may enhance positive affect. From a rider safety perspective, many equestrians report that horses, and especially ponies, become more reactive and potentially less tractable when fed more concentrates (particularly perhaps oats) and less forage. Fasting is associated with similar transient increases in both slow-wave sleep and REM sleep [30]. Some diets may better equip horses to cope with the stress of competition and fatigue. For example, foals receiving fat and fibre immediately after weaning, cantered less frequently, for shorter durations, and appeared to be more settled than those on a starch and sugar diet [31]. Meanwhile, high-starch diets alter equine faecal microbiota and increase behavioural reactivity [32]. It is not clear how high-energy diets change horses’ affective states. However, when these diets are accompanied by physical confinement, e.g., to stables or yards, it is clear that they are associated with an increase in the risk of post-inhibitory rebound [33] that can make individuals difficult to control.

Of the current sample, 37.40% showed at least some agonistic responses to one or more inter-specific threats (red cluster). The current findings indicate that horses can differentiate between other horses and seem to distinguish familiar dogs and other animals from unfamiliar dogs and other animals, in that these are in entirely distinct clusters. This highlights how much attention horses, as a prey species, need to attend to potential predators. It reminds us about the equine need to be able to predict the behaviour of others around them; something that is harder with an unfamiliar dog than a familiar one.

Finally, among the current sample of 1061 horses, 58.2% showed at least some agonistic responses to one or more intra-specific threat (blue cluster) situations. Given that these arise as interactions among horses, albeit sometimes when being ridden or driven, it is plausible that some of these may be more innate than the human-dependent responses that characterize other clusters. To that extent, they are more informative of inherent equine traits than of anthropogenic outcomes. Importantly, five of the seven contexts in this cluster are related to unfamiliar conspecifics. This is a reminder that horses take time to accept others as social group members. That said, to paraphrase William Butler Yeates, many horses behave as if “there are no strangers here; only friends you haven’t yet met”. Riding or driving in a group or in an arena occupies a different part of the dendrogram to any of the unfamiliar intra-specific contexts. So, the human management of horse behaviour in these contexts may dilute some of the signalling between horses and their agency to act on perceived threats. Similarly, it is striking that the unfamiliar horses approaching a focal horse are in a separate part of the dendogram from those being led/ridden beside or toward the focal horse.

Traditional equestrianism has been commonly associated with failures to consider horses’ social needs. Most equestrian activities require riders to overcome their mount’s innate liking for conspecific company and cannot afford unfamiliar horses to exercise agency when meeting under-saddle [34]. Denying social needs and normal greeting styles can lead to undesirable responses under-saddle. For example, while aggression toward conspecifics while under-saddling may mean that some horses cannot be ridden safely in company, unresolved separation-related distress may cause bolting [9]. The better socialised a horse is under-saddle, the less likely these responses are. In addition, full consideration of the social environment the horse occupies at home can reveal the relevance of this to agonistic intra-specific responses at exercise. Elements of E-BARQ that focus on how often home horses show signs of anxiety when left alone or when taken away from other horses will be the focus of a separate report.

It is important to acknowledge several limitations with the current approach. Since E-BARQ is anonymous and conducted online, participants are left to respond truthfully. So, some degree of respondent bias may be reflected in the current results. Clearly, some owners may have been tempted to report the behaviour of their horse more positively (or indeed negatively) than an unrelated observer might. Also, we ask respondents to respond with the listed option that is closest to their subjective assessment. This approach accepts that some of the options, for some respondents, that may be offered do not align with best practice or even logic. Furthermore, our use of equitation science mailing lists may have led to a form of selection bias, given that such individuals are interested in evidence-based ethical equitation [10]. It is probable that E-BARQ is more appealing to those who chiefly ride their horses rather than drive them. However, we have done our best to counter this prospect and remain especially keen to gather data on horses that are both ridden and driven, since these horses are of enormous value in understanding the interconnection between under-saddle and in-harness responses. The narrative description of undesirable behaviours within E-BARQ was brief but it is possible, that in doing so, we left some behaviours open to interpretation. Many owners are poor at recognising subtle signs of pain, confusion, or fear [4,35,36,37,38,39,40], so the numbers reported here probably represent a lower limit to the proportion of horses displaying agonistic behaviours. The current data probably reflect the observations of amateurs more than professionals, and thus reflect the proportion of both communities in the equestrian population. Similarly, the proportionally low distribution of male-to-female riders across equestrian disciplines may merit consideration when the interaction between equestrian disciplines preferred by females is explored in future studies. The authors advise the use of caution when interpreting data from a relatively small number of male respondents.

## 5. Conclusions

This study shows that owners report agonistic equine responses in clusters associated with locomotion under saddle, being saddled, disturbances in familiar environments, and both inter- and intra-specific threats. The circumstances that triggers within these clusters have in common reveal the strong probability that motivations change from one cluster to another. This finding will advance equine behavioural medicine, encourage analysis of the causal factors, and reduce the tendency to blame horses’ innate behaviour for unwelcome responses.

## Figures and Tables

**Table 1:** The 29 contexts of agonistic responses and the rotated components from a principal component analysis of the scores for these responses as they form six groups (highlighted according to the clusters in Figure 2). Following rotation, the variance of Component 3 exceeded that of Component 2 and Component 6 exceeded that of Component 5. The contextual triggers appear in rows and are shaded with the colours they were assigned from the cluster analysis. The clusters reveal horses whose thresholds for agonistic behaviour vary with: intra-specific threats (blue); inter-specific threats (red); extraneous disturbances in a familiar environment (green); saddling-up (yellow); and locomotory effort (purple).

	RC1	RC3	RC2	RC4	RC6	RC5
Verbally correction by owner on ground	0.07	0.45	0.25	0.13	0.37	0.27
Verbally correction when ridden/driven	0.14	0.57	0.18	0.08	0.23	0.26
Leg or whip cue when ridden/driven	0.14	0.69	0.04	0.04	0.11	0.21
Approach by owner in paddock	0.11	0.1	0.45	0.07	0.51	0.05
Approached by owner when tied	0.07	0.03	0.42	0.02	0.57	0.26
Approached by owner in stall	0.04	−0.04	0.49	0.05	0.6	0.18
Approached by stranger in paddock	0.16	0.16	0.85	0.12	0.05	0.06
Approached by stranger in stall	0.15	0.07	0.86	0.12	0.22	0.08
Approached by stranger when tied	0.17	0.11	0.85	0.14	0.18	0.12
Approached by owner when eating	0.14	0.15	0.12	0.06	0.76	0.1
Approached by owner carrying feed	0.14	0.26	−0.03	0.12	0.66	0.07
Saddle placed on back	0.19	0.16	0.13	−0.02	0.15	0.82
Girth done up	0.14	0.19	0.06	0.02	0.1	0.86
When hosed down	0.06	0.23	0.16	0.21	0.19	0.41
Approach by familiar dog	0.15	0.09	−0.02	0.78	0.12	−0.05
Approached by familiar animal	0.18	0.11	0.03	0.8	0.13	0.17
Approached by unfamiliar dog	0.23	0.1	0.22	0.78	0.03	−0.04
Approached by unfamiliar animal	0.25	0.13	0.25	0.77	0	0.1
Led toward unfamiliar horse	0.76	0.1	0.15	0.2	0.08	0.11
Approached by unfamiliar horse	0.75	0.08	0.12	0.18	0.04	0.11
Ridden/Driven toward unfamiliar horse	0.84	0.13	0.11	0.11	0.08	0.06
Led beside unfamiliar horse	0.86	0.12	0.07	0.14	0.07	0.1
Ridden/Driven beside unfamiliar horse	0.87	0.11	0.08	0.12	0.06	0.1
Ridden/Driven in a group	0.83	0.15	0.06	0.11	0.09	0.05
Ridden/Driven in arena with other horses	0.78	0.15	0.06	0.09	0.17	0
Lunged in a round pen	0.19	0.53	0.05	0.09	0.36	−0.04
Signalled forward under saddle	0.07	0.85	0.08	0.11	0.1	0.09
Signalled to canter under saddle	0.15	0.84	0.03	0.07	−0.02	0.04
Signalled to gait up under saddle	0.13	0.88	0.05	0.1	0.02	0.07
	RC1	RC3	RC2	RC4	RC6	RC5
Sum of Squares Loadings	5.12	3.9	3.14	2.76	2.56	2.03
Proportion of Variance	0.18	0.13	0.11	0.1	0.09	0.07
Cumulative Variance	0.18	0.31	0.42	0.51	0.6	0.67

## Data Availability

Data are contained within the article and supplementary materials.

## Supplementary Materials

A copy of the Equine Behavior Assessment and Research Questionnaire (E-BARQ) is available on Fig Share: https://doi.org/10.6084/m9.figshare.24115257; accessed on 1 September 2023.
